# Insights into the primate trade into the European Union and the United Kingdom

**DOI:** 10.1007/s10344-023-01681-3

**Published:** 2023-04-25

**Authors:** Magdalena S. Svensson, Vincent Nijman, Chris R. Shepherd

**Affiliations:** 1 Monitor Conservation Research Society, Big Lake Ranch, Canada; 2 Oxford Wildlife Trade Research Group, Oxford Brookes University, Oxford, UK

**Keywords:** Bushmeat, Legislation, Pets, Wildlife trade

## Abstract

Illegal and/or unsustainable trade is a major obstacle to effective primate conservation. The wildlife trade in the European Union (EU) and the United Kingdom (UK) is significant, but for many species, such as primates, the trade is poorly understood and sparsely reported. All EU countries are Party to the Convention on International Trade in Endangered Species of Wild Fauna and Flora (CITES); all primates are listed on Appendix I or II of CITES and are included on Annex A or B of Regulation (EC) No 338/97. We here combine data from several databases (CITES, UN Comtrade, TRAFFIC WiTIS) and seizure reports, to provide a narrative of the trade in primates into and within Europe. The legal import of live primates (2002–2021) amounted to 218,000–238,000 individuals (valued at US$ 869 million), with France, the UK, and Spain as the main importers and Mauritius, Vietnam, and China as the main exporters. Over 21,000 primate parts (trophies, skulls, bodies) were imported mainly from African countries, and UN Comtrade data suggests that ~ 600 tonnes of primate meat was imported mainly from Asia. The vast majority of live primates are either captive-born or captive-bred, and this proportion has increased over time. Reports of the illegal primate trade are far from complete, but the illegal trade of specific species or primate meat can have negative impacts of wild populations of already imperiled species. Stronger policies and more effective enforcement in consumer countries, such as the EU, would also aid in, and garner support for, better protecting primates in primate range states.

## Introduction

Of the 522 currently recognised primate species, 60% are threatened with extinction, and nearly 75% face population declines (Estrada et al*.* 2017; IUCN Primate SG 2021). Domestic and international trade in primates is considered a major impediment to primate conservation globally (Duarte-Quiroga and Estrada 2003; Nijman et al. 2011; Blair et al. 2017; Estrada et al*.* 2017; LaFleur et al. 2019; Scheffers et al. 2019; Norconk et al. 2020). Primates are traded for a number of purposes, including use in research, as pets, food, ingredients in traditional medicines, for entertainment, trophies, and for collections (Nijman et al. 2011; Linder et al. 2013; Estrada et al. 2017). The global trade of live primates, both legal and illegal, has been estimated at US$ 138 million annually (Norconk et al. 2020).

Primate trade to Europe has a long history, reaching back to 50 BC when primates were imported as pets to Ancient Rome (Morris and Morris 1966). In medieval times, primates were among the most popular pets with Europeans who could afford them, and primate furs were imported and traded for fashion (Morris and Morris 1966; Walker-Meikle 2012). African primates were widely available in Europe from the twelfth century onwards (Veracini 2017), and from the sixteenth century, Neotropical primates were traded as exotic pets for European aristocrats (Urbani 1999, 2007). European countries continue to be the top importers of primates, with the United Kingdom (UK) and France reportedly being the fourth and fifth biggest importers of primates globally in 2016 (Nijman and Healy 2016). In 2017, the Observatory of Economic Complexity reported that Europe was one of the main importers of primates, representing 31% of the primate imports globally (Norconk 2020). Contemporary data are lacking, but based on declared import data, Engler and Parry-Jones (2007) estimated that in the year 2005 the European live primate trade had a monetary value of €15 million (corrected for inflation this equals €21.2 million in 2023).

Seizures of illegally imported wildlife reported by European Union (EU) member states are reportedly increasing, rising from 5644 records reported in 2017 to 6441 in 2019, most of which were reported by France, Germany, the UK, Spain, and the Netherlands (TRAFFIC 2020, 2021). These seizures were of wildlife mainly intended for medicinal purposes (derived from both plants and animals), but only a minority of the seizures involved live mammals (TRAFFIC 2021). However, it is known that bushmeat trade into the EU is occurring on a large scale, including primates, but the exact figures are not currently known. As an example, an estimated 40 tonnes of bushmeat is illegally imported via Swiss airports, and 270 tonnes via Charles de Gaulle airport in France annually (Wood et al. 2014; Mowbray 2017). It is likely that other European airports and borders would have similar numbers (Wood et al. 2014). The European bushmeat market represents a lucrative endpoint for illegal bushmeat trade (e.g., from Africa), where high prices are paid for increasingly rare African species, making the illegal international trade worth the potential, but often low, risks (Mowbray 2017).

The trade in primates as pets into Europe is also ongoing; for example, it is believed to have caused a decline of 80% in the wild population of Barbary macaques (*Macaca sylvanus*, Encap 2012; van Uhm 2016a). Whilst keeping primates as pets is fully banned in some EU member states (e.g., Bulgaria, Estonia, Hungary, Italy, Latvia, Lithuania, the Netherlands, and Portugal), in other European countries, keeping certain species of primates as pets can be legal; this includes Austria, Belgium, Denmark, Poland, and the UK (Food and Agricultural Organization of the United Nations (FOALEX) 2022).

Although throughout the EU and UK primates are used only in exceptional circumstances for laboratory experiments, where no alternative methods are possible and no other species may suffice for the purposes of the studies, studies on great apes are effectively banned. In 2018, 8583 primates were used for scientific purposes in the EU (European Commission 2019); these are first-time uses and more individuals were involved in reuses. The most common species by far was the long-tailed macaque (*M. fascicularis*) with marmosets and tamarins being the next most commonly used species. For those primates that were used for the first time for scientific research in 2018, the majority were sourced from self-sustaining colonies (29%) or as second- or higher generation captive-bred (56%), and none was reported as sourced from the wild.

The *Declaration of the European Parliament on Primates in Scientific Experiments* was established in 2007 (EU Parliament 2007). This Declaration called for an immediate restriction on the use of great apes and wild-caught monkeys, and to phase out the use of all non-human primates, replacing them with alternatives. However, in the UK alone, it was reported that in 2019, the number of import permits for primates and their derivatives imported for laboratory animal experiments had almost tripled from previous year, to over 6700 permits (Dalton 2020).

All primate species are listed by the Convention on International Trade in Endangered Species of Wild Fauna and Flora (CITES), either under Appendix II, meaning commercial trade is regulated, or in Appendix I, which generally precludes commercial trade. CITES is ratified by all EU member states and the UK, as well as by the European Union itself (Policy Department of the European Parliament 2016; CITES 2022a). CITES regulations are implemented within the EU legislation through Council regulations EC No 338/97 and EC No 865/2006 (European Commission 2010). The EU Enforcement Action Plan (*Commission Recommendation 2007/425/EC*) recommends that EU member states develop inter-regional collaboration to combat illegal wildlife trade by building links with other regional and sub-regional initiatives (Nijman and Shepherd 2009). According to the CITES National Legislation Project, all EU countries and the UK fall in Category 1, meaning that each countries’ legislation is believed to generally meet the requirements for implementation of CITES (CITES 2022b). Furthermore, the EU has its own EU Wildlife Trade Regulations, where all animal and plant species are listed under four annexes, including live and dead specimens and their derivatives, to aid in monitoring import levels (European Union 2022). In general, these annexes follow the appendices of CITES, with Annex A being equivalent to CITES Appendix I and Annex B being the equivalent to CITES Appendix II. However, for primates, about 30 Appendix II species are included on Annex A, including black colobus (*Colobus satanas*), several langurs (*Trachypithecus* spp.), titi monkeys (*Callicebus* spp.) and all tarsiers (*Carlito syrichta*, *Cephalopachus bancanus*, and *Tarsius* spp.). As such, as a group, primates are well covered in terms of import and export regulations.

While considerable work has been carried out on the trade in primates in range countries, for example, in Southeast Asia and Africa (Nijman 2010; Shepherd 2010; Sogbohossou et al. 2018; Estrada et al. 2019; Svensson et al. 2021), little work has been done to understand the scale and dynamics of the trade in consumer countries. The EU, with its 27 member states, and the UK, are known to be major consumers of wildlife from around the world (van Uhm 2016a; Halbwax 2020), yet little work has been done to quantify and qualify the trade in species groups such as primates. There is a dearth of published evidence on source countries, the species involved, and the purposes for which primates are traded in consumer countries. There is also an absence of readily available information illustrating the legality of the trade and the efforts made to prevent illegal trade in primates trafficked to the EU and UK.

With our study, we aim to quantify and qualify the trade in primates to the EU and UK, currently and over the last 20 years, with a focus on both the legal and the illegal trades to support efforts to eliminate the illegal trade of primates. By doing this, we hope to provide baseline knowledge on the trade in primates entering the EU and the UK, determining which species are involved, where they are coming from, what they are traded for, and what portions of this trade are legal or illegal. We further hope this baseline data will aid future monitoring, analysis and management recommendations to better regulate this trade and support enforcement efforts and policy interventions.

## Methods

### Legal trade

We downloaded data, in December 2022, on the import of primates to the EU and the UK from the CITES trade database (http://trade.cites.org/) for the period 2002–2021 (data from 2022 were not yet available).

Although the UK left the EU in January 2020, we have decided to include it in our study. The data collected is mainly from before January 2020, and the EU legislation regarding wildlife trade and import controls remains incorporated in UK domestic law as “retained EU law” under the European Union (Withdrawal) Act 2018, and will remain in force, without amendment, apart from corrections to make the EU legislation fully operable (APHA 2021).

We established the number of imported live and dead primates, as well as the main source and destination areas, assessing the proportion of wild-caught vs captive-bred or captive-born individuals. Regarding specimens in the CITES database, it is possible to overestimate the number of individuals, as specimens are defined as any readily recognizable part or derivative of the animal (we use the definition of specimen as described by www.CITES.org). To avoid over-counting, we excluded specimens where it was specified that the import was in metric volume units. We restricted dead individuals to trophies, bodies, and skulls to avoid possible double-counting (a skull and a skin imported on two separate occasions could be derived from the same individual). To prevent double-counting, we checked all re-exports (when an individual is exported by one country after it has been imported from another). Reported import and export figures did not always coincide; therefore, we crosschecked the numbers and included the largest reported numbers by comparing figures from importing and exporting countries. The CITES trade database is conservative in the taxonomy employed, and taxonomy is listed as it was at the time of reporting. We, therefore, where possible, refined the taxonomy of primate species listed based on current knowledge of distribution patterns.

The UN Comtrade database holds records of imports and exports of primates and primate derivatives, including declared monetary values. It does not provide information on the species of primates that are traded internationally, although in some instances this can be inferred from other data (Hansen et al. 2022). For the same two decades, we downloaded the reported import records (HS codes 010611, 021091 and 020830, covering live primates and primate meat) from the 27 EU countries and the UK to provide an estimate of the monetary value of this trade. We corrected the monetary values for inflation to December 2022. We checked the number of live primates against their reported mass, and entries of primates of a mean weight of less than 300 g were considered erroneous and were omitted. We also omitted three entries that were more than two standard deviations above the average number of individuals and were therefor considered erroneous; the same was done for meat. Further entries with just weight or monetary value reported were deleted. In terms of value, the mean of all live primates reported in the UN Comtrade equaled US$ 2684, which coincides well with the commercial value of the primate trade reported by Hansen et al. (2022); we considered live primates traded for less than US$ 100 to be erroneous, and these were omitted. Likewise, the mean price for all primate meat equaled US$ 33/kg; entries of less than US$ 2/kg or more than US$ 200/kg were omitted.

### Illegal trade

The CITES trade database only holds records of reported, and therefore legal, international trade. To give an indication of the scale of the illegal trade in primates entering the EU and the UK and to highlight species in demand, as well as identify routes and hotspots, we additionally used the seizure data sourced from CITES management authorities and other government agencies and downloaded data from the TRAFFIC WiTIS seizure database. In this database TRAFFIC has gathered open-source wildlife seizure and incident data reported to them, but in a somewhat opportunistic way and not evenly over all countries. We also systematically scoured the Internet, using Google, for seizures of illegal wildlife trade reported in the media using keywords, cutting off our search effort at two pages for each combination of keywords searched (Stringham et al. 2021). We chose to focus our searches in the majority EU languages, including English, French, Spanish, German, Dutch, and Swedish. There are 24 official language in the EU, but often records were reported in more than one language and we anticipate that our search strategy would have captured the majority of relevant records. No local authorities were contacted.

We are fully aware that reported seizure data, especially when sourced from the media, does not provide a complete dataset for analysis; however, it does provide indications of the trade dynamics and examples of illegal wildlife trade scenarios, especially when looking at data over such an extended period (Cheng et al. 2017; Siriwat and Nijman 2018). Using data reported in the TRAFFIC WiTIS database, and reports found through our own scouring of the media reports, we also aimed to conduct a preliminary assessment of prosecutions.

## Results

### Legal trade in live animals

The total number of live primates reported to the CITES trade database as having been imported into EU/UK countries over the 20 year period was 218,189 individuals. The main importers were France (63,196 individuals), UK (57,586), Spain (44,688), the Netherlands (23,243), and Germany (18,061). The main exporters were Mauritius (109,606 individuals), Vietnam (43,854), China (40,135), Barbados (5176), and the Philippines (3574). There was some trade within the EU and the UK, between UK and Spain (1644 individuals), and between Hungary and the Netherlands (133). In all at least 139 identified primate species were imported into the EU and the UK. The main species were long-tailed macaques (*Macaca fascicularis*, 196,229 individuals), rhesus macaques (*M. mulatta*, 8870), and green monkeys (*Chlorocebus aethiops*, 5126). Live primates, where purpose and source was recorded, were imported to the EU and the UK mainly for medical (46.83% of all reported imports), commercial (28.67%), or scientific (22.84%) purposes, and the majority were sourced from populations bred or born in captivity (95.50%).

**Fig. 1:**
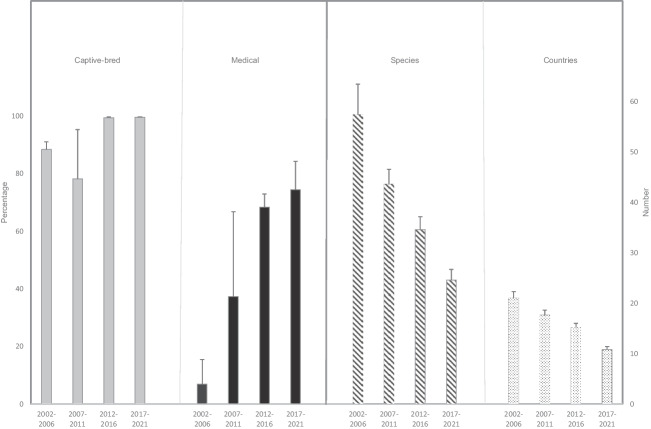
Trends in imports of live primates (mean+1 standard deviation) into the European Union and the United Kingdom (2022–2021), looking at proportion sourced from captive population and traded for medical purposes, as well as the average number of species and number of EU (including the UK) countries importing live primates, as reported to the CITES database

Concerning trends over the last 20 years, it is evident that the majority of live primates imported into the EU and the UK are either captive-born or captive-bred, and this has reached nearly 100% over the last decade. The proportion of primates being traded live for medical purposes has increased over the last two decades (although in the past some of primates imported for biomedical research may have entered the EU under scientific or commercial purpose codes). There has been a steady decline in both the number of species and the number of countries that have imported live primates (Fig. 1).

The total number of live primates reported to the UN Comtrade database as having been imported into the EU and the UK over the 20 year period was 237,753 individuals. The main importers were France (123,798 individuals), Spain (31,349), and the UK (23,555). The main exporters were Mauritius (77,529 individuals), Vietnam (29,365), and China (incl. Hong Kong, 19,143). The UN Comtrade database indicates a considerable within-EU trade with, for instance, relatively large numbers traded between France and Spain (27,449 individuals), the Netherlands and Italy (5204 individuals), and Spain and Germany (4127 individuals). The total declared monetary value of the live primate trade (including re-exports) over the two decades, when corrected for inflation, amounted to US$ 869 million.

### Legal trade in primate parts

The CITES trade database contains records of 21,060 primate items (45.40% skulls, 42.08% trophies, and 12.52% bodies) over the 20-year period. The skulls were mainly from baboons (*Papio* spp., 7228 individuals) and vervets (*Chlorocebus* spp., 1939); trophies were mainly of the same two species, *Papio* spp. (7487) and *Chlorocebus* spp. (1261); whilst most of the bodies were marmosets (*Callithrix* spp., 1726, mainly traded for scientific purposes) and baboons (167). The main importers were Germany (parts from 7390 individuals, of which most were skulls), Spain (2319, of which most were trophies), and Denmark (1545, of which most were trophies). The main exporters were South Africa (7968, of which most were skulls and trophies), Namibia (6700, of which most were skulls and trophies), and Brazil (1892, of which most were bodies). For the primate parts where purpose and source was recorded, skulls, trophies, and bodies were imported to the EU and the UK mainly as hunting trophies (64.82% of all reported imports) or for scientific (12.53%) or commercial (11.96%) purposes. The majority were sourced from wild populations (96.29%) of which 43.03% were imported as trophies, mainly from South Africa (3608 individuals) and Namibia (2786).

**Fig. 2:**
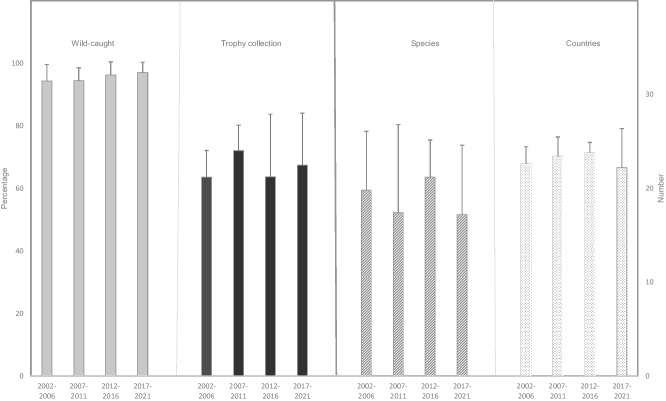
Trends in imports of primate parts (mean+1 standard deviation) into the European Union and the United Kingdom (2022–2021), looking at proportion sourced from wild population and traded for trophy collection purposes, as well as the average number of species and number of EU (including the UK) countries importing primate parts, as reported to the CITES database

Concerning trends over the last 20 years, it is evident that primate parts imported into the EU and the UK are continuously sourced from wild populations. About two-thirds the trade in primate parts is reported for trophy collection purposes, and this has remained stable over the last two decades. Both the number of species and the number of countries that have imported live primates has also remained relatively stable (Fig. 2).

The UN Comtrade database contains records of the trade in primate meat. In total, over the two decades, 6251 tonnes of primate meat was reportedly imported into individual EU countries, but almost all of this referred to trade within the EU. Restricting this to exports from primate range countries, it totals 589 tonnes over the 20-year period. The main exporters were Indonesia (401 tonnes), Taiwan (132 tonnes), and China (31 tonnes). The total monetary value of the primate meat in trade over the two decades, when corrected for inflation, amounted to US$ 3.11 million.

**Fig. 3:**
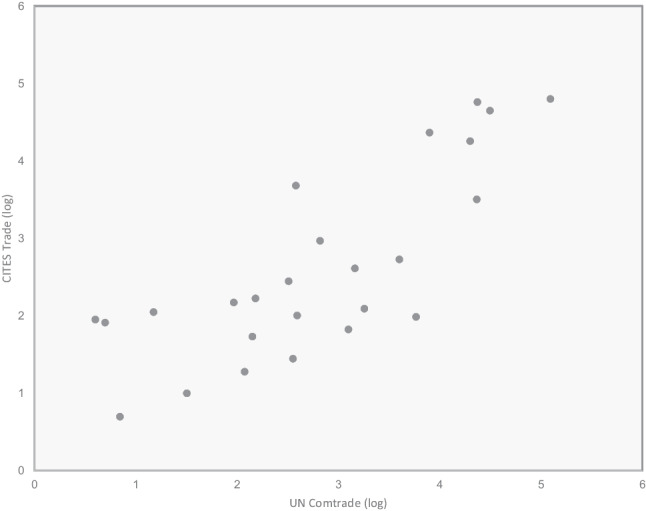
Concordance between the CITES trade database and the UN Comtrade database on the number of live primates imported into 25 EU countries over the last two decades [Pearson’s *R* = 0.7875, *R*.^2^ = 0.6202, *P* = 0.00001]

While the numbers between the CITES trade database and the UN Comtrade database showed quite some discrepancies for individual countries, overall, there is a good degree of concordance at the EU level (Fig. 3).

### Illegal trade

Concerning seizure of illegally traded primates, using the TRAFFIC WiTIS database and media reports, 58 seizures were recorded. Most seizures were reported in the UK (27 reports), France (6), Spain (5), and the Netherlands (4), but there were also reports from Germany, Sweden, Belgium, Italy, Poland, Greece, Hungary, Ireland, and Portugal. Regarding the number of individual primates seized, German authorities reportedly seized 569 primates, followed by the UK which has seized more than 76 primates, and Spain (33). Often reports of seizures did not report actual numbers, so these are minimum esitmates.

Many of the seizures were made within country, not when entering a country, and the origin was not always reported. For the seizure reports on primates being illegally brought into the EU and the UK over the past 20 years where the origins were included, the majority arrived from Africa, with 22 seizures involving more than 45 individual primates. Most of these seizures involved dead primates or parts of primates (61%), and mainly coming from Cameroon and Madagascar. From Asia, all of the primates seized were dead/parts, and mainly from Indonesia (55%).

In 19 of the primate seizure reports, the species was not identified or reported. Out of the remaining 39 seizure reports, the species with the highest demand on the illegal market were long-tailed macaques. This was due to a seizure from the United States to Germany for scientific purposes and involved 572 individual macaques. The second most seized primates were baboons, with 13 individuals, of which all were seized as skulls or parts. African great apes were seized on four occasions, all dead/parts (two chimpanzees (*Pan troglodytes*), one gorilla (*Gorilla* spp.), and one just reported as great ape). Other primates that were illegally traded as parts/meat/dead were langurs (*Trachypitchecus *spp.), grivets (*Chlorocebus aethiops*), other macaque species, orangutans (*Pongo *spp.), and red colobus (*Piliocolobus *spp.). The most commonly seized live primate was marmosets (8 individuals), followed by Barbary macaques (3), capuchins (*Cebus* spp., 2), gibbons (*Hylobates* spp., 2), pygmy loris (*Xanthonycticebus pygmaeus*, 1) and ring-tailed lemur (*Lemur catta*, 1). Five guenon species were seized (*Cercopithecus *spp.), live and dead, but it was not indicated how many of each.

Out of the 58 reports of illegal seizures found in this study, we were able to find prosecution information for 36 of the incidents. Most commonly (in 15 incidents), the outcome of the incident was that the primates were simply confiscated. In eight incidents, the person(s) got a prison sentence, with the imprisonment length ranging from 3 to 66 months (both extremes in the UK). Four of these prison sentences were in the UK, and the others in Greece, France, Spain, and the Netherlands, and all between 2013 and 2021. In seven incidents the person(s) got a fine, sometimes in addition to imprisonment (reported in four incidents), and the fines ranged between US$ 316 and US$ 244,453. The lower fine was given in Sweden and the highest in Spain. All reported fines were between 2013 and 2021.

## Discussion

The EU, the UK included, is considered one of the major hubs in terms of wildlife trade, being the top global importer of wildlife and their derivatives by monetary value and ranking as the third-largest wildlife trade hub in the world in terms of consumption and transit (Engler and Parry-Jones 2007; Sollund and Maher 2015; Halbwax 2020). Our data, obtained from various independent sources, indicate that the EU ( including the UK) also is an important importer of primates, both alive and their parts. The data from the CITES trade database and the UN Comtrade database showed a good degree of consistency, especially where it pertained to the trade in live primates. Data on trade in primate meat could only be obtained from the UN Comtrade database, and here the very large amount of primate meat being traded between EU countries makes us believe that this is because of incorrect use of HS codes rather than the existence of a substantial Europe-wide trade in primate meat. The EU and the UK have a big role to play in clamping down on illegal wildlife trade to prevent the decline in wildlife species and the spread of zoonotic disease (Halbwax 2020). The scale of the primate trade may be a cause of concern for some wild primate populations, as exemplified by the Barbary macaques (Encap 2012). Van Uhm (2016b) reported that Barbary macaques were the most seized illegally traded live animal in Europe from 2001 to 2010. Morocco, where these macaques range, has emerged as one of the main wildlife trafficking gateways from Africa to Europe (Van Lavieren et al. 2016), and it is concerning that our findings indicate that Barbary macaques are still being illegally imported into Europe.

In our findings on the illegal primate trade coming into the EU and the UK, we saw most of the primates being imported from African countries, mainly Cameroon and Madagascar, as meat or parts. This mirrors the findings reported by Mundy-Taylor (2013), where seizures of all wildlife species traded between 2007 and 2011 showed that the majority of primate seizure records were exported from Namibia, South Africa, Democratic Republic of Congo, Cameroon, and Central African Republic. Most of the seizures reported by Mundy-Taylor (2013) involved skulls or whole dead primates for consumption. Most reports we found of dead primates being legally imported were also from Africa, mainly live (from Mauritius) or trophies (from South Africa and Namibia). This was also mirrored in the illegal trade we report on, where the main part of seizures was of dead primates/parts from the African continent. Large amounts of bushmeat are as mentioned being seized at European borders and airports (Wood et al. 2014; Mowbray 2017), and bushmeat has been found to be sold in restaurants and markets. For example, endangered primate species, such as red-tailed monkey (*Cercopithecus ascanius*) and De Brazza’s monkey (*C. neglectus*), were identified in Brussels (Bradshaw 2018; Lu 2020).

The most commonly legally traded dead primates in our study were also from the African continent, with baboons being the most imported primate as trophies, bodies, and skulls. However, when it comes to legally imported live primates, the Asian primates, macaques, are the most in demand, although the majority were reportedly captive bred in Mauritius, followed by China and Vietnam. We found these macaque species being heavily overrepresented in both the legal and illegal trades, and continue to be legally traded in significant numbers. The same has been indicated in previous studies. Between 2010 and 2014, long-tailed macaques were one of the ten most traded live species worldwide (D’Cruze and Macdonald 2016).

The COVID-19 pandemic had a significant effect on global trade, including that of primates. For instance, in February 2020, China, until then the largest exporter of live primates mainly for biomedical purposes, banned the export of primates (Koh et al. 2021). With demand for live primates not being diminished, this may have led to significant shifts in the sourcing of primates.

We did not record many seizures of illegally imported primates into countries such as Poland, but it has been found that primates are among the most traded groups of wildlife in the Polish online trade (Paquel 2016). This was mainly for the pet trade and mostly included primates listed on CITES Appendix II, such as marmosets, Barbary macaques and patas monkeys (*Erythrocebus patas*). Monitoring the trade in pet primates is vital to reduce the risk of zoonosis. Primates act as reservoirs for a fifth of the diseases that affect humans, and their close phylogenetic relationship to humans amplifies the risk of transmission (Wolfe et al. 2007).

Prosecution information was not always reported, so we were unable to measure prosecution rates. However, our findings indicate that punishments are seldom handed out, and if so, the fines or imprisonment lengths tend to be minimal.

## Conclusion

The clandestine nature of the illegal wildlife trade, and insufficient resources at EU boarders, as well as loopholes in enforcement policies and law penalties, makes the exact quantities of trade hard to measure (Wyatt and Cao 2015; Sollund and Maher 2015). It is however believed that wildlife trade into Europe is increasing. According to Evans (2018), the UK Border Force reported a nearly 650% increase in seized wildlife between 2011 and 2017, which could be due to either an increase in trade, or an increase in detection and awareness of the illegal trade. It is thought that the legal wildlife trade in regions such as the EU is driving up a parallel illegal trade (Cook et al. 2002). Further investigation into illegal trade chains leading into the EU and the UK is required, to provide detail to support international enforcement efforts and conservation interventions to be carried out. We recommend further, more in-depth research evaluating the scope and scale of primates in the EU bushmeat trade, as well as to look at the potential risks posed by primates used for scientific purposes in the EU and the UK. As long as this illegal trade of primate persists wild populations of primates will continue to decline. While there is a need for enforcement agencies to increase their vigilance in the EU and the UK, efforts should be made to trace illegal supply chains back to the source countries and to collaborate with authorities there to end this illegal trade, as well as reduce the demand in Europe.

## Data Availability

The datasets generated during the current study are available from the corresponding author on reasonable request.
